# Ohio State Dental Society

**Published:** 1874-01

**Authors:** 


### OHIO STATE DENTAL SOCIETY.

The sixth annual meeting of this Society was held in Columbus, on the 3d of Dec. and the two succeeding days.

There was a large attendance, about one hundred including members and visitors.

It was one of the best meetings of that Society ever held. Many subjects of interest were presented and discussed—many of the members taking part. Several interesting and instructive papers were read. The Judiciary Committee appointed at the last annual meeting to obtain statistics of the profession in Ohio, made a very interesting report: which though not entirely complete contained much valuable information. There was much in it to encourage, and somewhat to discourage.

The profession in the State is being rapidly improved. Some who three or four years ago had very little knowledge of either the science or the art of the profession, and were necessarily very inferior operators, have by close study and application made very marked improvement, and so far familiarized themselves with the better methods of practice, as to be the means of great good to those who may be placed in their care. professionally.

Some others, despairing of being able to make respectable attainments, abandoned the profession and engaged in pursuits more within the range of their ability. And still others, though continuing in the profession, found it convenient and desirable to migrate to more congenial places, to states where there are no legal disabilities or restrictions ; where any one with a pupilage of a few weeks and an unlimited amount of assumption, audacity and impudence may go forth to practice his quackery and charlatanism upon any and all who may consent to become his victims.

Now all this comes through influence of the State Society, and the legal enactments regulating the practice of dentistry in Ohio, secured by the effort of this Society.

Many other interesting items are presented in this report, to which we will not now refer as the report will soon be published.

The Society enjoyed the unexpected pleasure of the presence and assistance of Prof. W. H. Atkinson, of New York, who as a teacher has no superior and few, if any, equals in this or any other country. In his teaching, however, he pursues a course distinctly his own—one that scarce any one else need have even a shadow of a hope of imitating. The Doctor congratulated the Society in very warm and glowing terms.

It was also a matter of great pleasure to all to have Dr. Watt present. He had been detained for one or two years from the meetings by sickness. The Doctor for several years has been a great sufferer, and most of the time not able to be from home at all. He surprised all of his friends and perhaps himself by the exhibition of as great, if not greater, mental powers than ever before.

The Board of Examiners, acting under the law, had before them quite a number of applicants and several of them old practitioners who were not amenable to the law, but from a just and laudable desire to place themselves in a correct position in the profession, submitted to an examination, that they might have whatever of influence and prestige would accrue from a certificate of professional qualification, given upon a thorough examination.

There was quite a large number added to the membership of the Society. The details and particulars in all these matters will appear in full in the official minutes which we hope to publish in the February No. of the REGISTER. The Ohio State Dental Society is a powerful agent for good to the profession in the State, and in the aggregate far more to the public.

